# The Immuno-Modulation Effect of Macrophage-Derived Extracellular Vesicles in Chronic Inflammatory Diseases

**DOI:** 10.3389/fimmu.2021.785728

**Published:** 2021-12-16

**Authors:** Yi Xing, Xun Sun, Yiming Dou, Min Wang, Yanmei Zhao, Qiang Yang, Yanhong Zhao

**Affiliations:** ^1^ Department of Orthodontics, Hospital of Stomatology, Tianjin Medical University, Tianjin, China; ^2^ Department of Spine Surgery, Tianjin Hospital, Tianjin University, Tianjin, China; ^3^ Institute of Disaster and Emergency Medicine, Tianjin University, Tianjin, China

**Keywords:** macrophage-derived extracellular vesicles, immunomodulation, chronic diseases, therapeutic strategy, inflammation

## Abstract

As natural nanocarriers and intercellular messengers, extracellular vesicles (EVs) control communication among cells. Under physiological and pathological conditions, EVs deliver generic information including proteins and nucleic acids to recipient cells and exert regulatory effects. Macrophages help mediate immune responses, and macrophage-derived EVs may play immunomodulatory roles in the progression of chronic inflammatory diseases. Furthermore, EVs derived from various macrophage phenotypes have different biological functions. In this review, we describe the pathophysiological significance of macrophage-derived extracellular vesicles in the development of chronic inflammatory diseases, including diabetes, cancer, cardiovascular disease, pulmonary disease, and gastrointestinal disease, and the potential applications of these EVs.

## Background

Extracellular vesicles (EVs) are natural phospholipid bilayer-derived particles expressing specific surface markers (e.g., tetraspanins, Alix, and TSG101) secreted by cells into the extracellular space (1). EVs have been isolated from various types of cells, tissues, and even bodily fluids (2). They are categorized mainly as exosomes (~40–160 nm diameter), microvesicles, and apoptotic bodies (~50 nm to 1 μm diameter) (3, 4). Initially, EVs were considered waste released by cells (5). More recently, their roles in cell–cell interactions have been identified (6, 7). Various cargos in the form of nucleic acids, proteins, lipids, and metabolites are transferred by EVs to recipient cells, thereby influencing the biological functions of those cells. EVs are highly heterogeneous and dynamic, depending on the parental cell source and microenvironment (3). Due to their unique characteristics and properties, EVs are critical mediators of various physiological and pathological processes including the immune response, cell proliferation and migration, tumor invasion, and metastasis (8–10). Furthermore, they are used as diagnostic tools and as therapeutic delivery systems carrying biological factors and/or drugs (11–13).

Macrophages (Mφ) are derived from monocytes in the bone marrow and are involved in specific and nonspecific immunity of the body. In nonspecific immunity, the biological functions of Mφ include removing dead cells and cellular debris, and presenting antigens for recognition. In specific immunity, activated Mφ have immunomodulatory functions by secreting cytokines. Moreover, they play a major role in antigen presentation and initiation of the immune response (14). Mφ are divided mainly into two phenotypes: classically activated M1 (M1Mφ) and alternatively activated M2 (M2Mφ) (15). Mφ play essential roles in the microenvironment and are also regulated by that microenvironment. Phenotypic polarization of Mφ has a dynamic influence on the balance between inflammation and tissue repair. Moreover, the functions and properties of EVs secreted from Mφ (Mφ-EVs) are influenced by Mφ polarization, with different phenotypes of Mφ-EVs being involved in diverse biological processes under various physiological and pathological conditions (16).

Inflammation is defined biologically as the response of the body’s immune system to a stimulus. Mainly caused by various pathogens and tissue damage, it plays an important role in tissue repair and is considered a protective response of the organism (17). Activated Mφ dominate the histopathology of chronic inflammation and can amplify the inflammatory response by mediating the release of inflammatory mediators (18). In 2010, the WHO stated that chronic diseases such as cardiovascular disease, diabetes, cancer, and chronic respiratory disease account for approximately two-thirds of global deaths (19). To date, several studies have confirmed the link between chronic inflammation and chronic disease, although the exact mechanisms are still not clear (20–22). Inflammation not only plays an important role in host defense mechanisms but also greatly contributes to the pathological process of chronic diseases. Therefore, targeting inflammation is a promising strategy for improving and treating chronic diseases.

In this review, we summarize the crucial role of Mφ-EVs in the pathogenic mechanisms of chronic diseases such as atherosclerosis, diabetes, cancer, lung-related disease, cardiovascular-related disease, and gastrointestinal-related disease. In addition, therapeutic strategies based on Mφ-EVs are discussed, as well as the challenges associated with their application.

## Mφ-EVs

**Figure 1:**
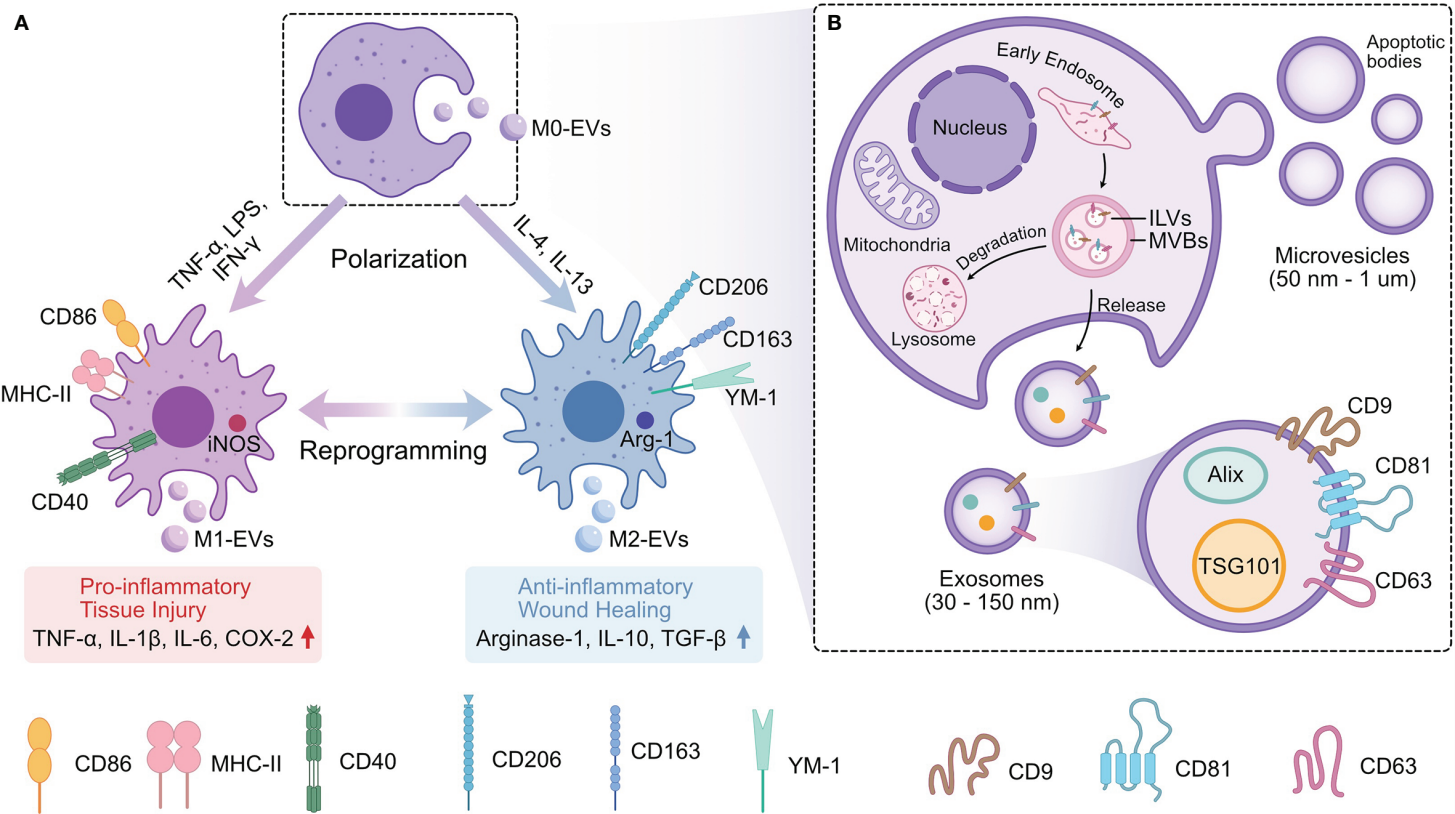
The Relation of Mφ and Mφ-EVs. **(A)** EVs derived from different phenotypes of Mφ (M0-EVs, M1-EVs, and M2-EVs) have different properties and biological functions. **(B)** EVs are released from Mφ by either secreting microvesicles and apoptotic bodies extracellularly through plasma membrane fusion or releasing exosomes by endosomal pathway. Mφ, Macrophages; EVs, Extracellular vesicles; Mφ-EVs, Macrophage-derived exosomes; TNF-α, Tumor necrosis factor alpha; LPS, Bacterial lipopolysaccharide; IFN-γ, Interferon gamma; IL-1β,4,6,10,13, Interleukin-1beta,4,6,10,13; CD40,86, 163,206, Surface markers in Mφ; MHC-I, Major histocompatibility complex II; iNOS, Inducible nitric oxide synthase; YM-1, Chitinase-like protein; Arg-1, Arginase-1; ILVs, Intraluminal vesicles; MVBs, Multivesicular bodies; Alix, Apoptosis-linked gene 2-interacting protein X; TSG101, Tumor susceptibility gene 101; CD9, 63,81, Tetraspanins; COX-2, Cyclooxygenase-2; TGF-β, Transforming growth factor-beta.

EVs are vesicles derived from the phospholipid bilayer released by cells (23), including various subtypes of nanoscale-to-microscale particles. They transmit information that helps regulate recipient cells (**Table 1**). Furthermore, they retain the biological properties of the parental cells (71). Thus, different phenotypes of Mφ-EVs play different roles in different pathological conditions (**Figure 1**).

**Table 1:** The cargos transmitted by Mφ-EVs to recipient cells.

EVs Source	Precondition of Macrophages	Disease model	Cargos	Mechanism	Reference
M2-EVs	–	Hepatocellular carcinoma	Integrin α_M_β_2_ (CD11b/CD18)	Promote invasive and metastasis of hepatocellular carcinoma cells *via* activating MMP‐9	(24)
Mϕ-EVs	Induced by LPS	Acute liver injury	Differentially expressed proteins like IL1rn, Gbp2	Activate the NLRP3 and NOD-like receptor signaling pathway	(25)
M2b-EVs	–	Colitis	CCL1 chemokine	Interact with CCR8 to increase IL-4 expression and Treg percentages	(12)
Mϕ-EVs	Treated with endotoxin and nigericin	Autoimmune diseases	The immune response-related proteins	Activate NF-κB signaling pathway	(26)
TAM-EVs	Reprogramed glioblastoma-derived EVs	Glioblastoma	Arginase-1	Promote tumor growth	(27)
Mϕ-EVs	Stimulated with angiotensin II	Bleomycin -induced lung fibrosis	Angiotensin II type 1 receptor	Activate TGF-β/smad2/3 pathway	(28)
Mϕ-EVs	Exposed to cigarette smoke condensate	HIV-1	Catalase	Protect U937 cells from oxidative stress and HIV-1 replication	(29)
Mϕ-EVs	Exposed to silica	Silicosis	BIP, XBP1s and *P*‐eIF2α	Induce endoplasmic reticulum stress	(30)
Mϕ-EVs	High glucose–treated	Diabetic nephropathy	TGF-β1 mRNA	Activate TGF-β1/Smad3 signaling pathways	(31)
Mϕ-EVs	Oxidized LDL-stimulated	AS	EVs transfer	Attenuate the growth and tube formation of endothelial cells	(32)
Mϕ-EVs	Treated with Shiga toxin 2a toxoids	Cells death	Globotriaosylceramide (Gb_3_), IL-1β and IL-8 mRNAs	Activate stress-associated MAPKs and induce ER stress in Gb3-expressing cells	(9)
Mϕ-EVs	–	–	Integrin β1	Promote internalization of integrin β1 in primary HUVECs, make the internalized integrin β1 accumulate in the perinuclear region and not recycled back to the plasma membrane.	(33)
Mϕ-EVs	–	Breast adenocarcinoma	Human a disintegrin and metalloproteinase 15	Enhance binding affinity for integrin αvβ3 in an RGD-dependent manner and suppress vitronectin- and fibronectin-induced cell adhesion, growth, and migration	(34)
Mϕ-EVs	Treated with interferon-α or not	Viral infection	Differentially expressed proteins	Be involved in two of the top biological process categories: “Defense response to virus” and “Type I interferon signaling pathway”	(35)
Mϕ-EVs	Exposed to silica	Silicosis	SPP1 protein	Phagocytosed by fibroblasts and generate corresponding myofibroblasts	(36)
Mϕ-EVs	–	–	Leukotriene B(**4**)	Produce chemotactic eicosanoids and induced granulocyte migration in the present of Ca (2^+^)-ionophore and arachidonic acid	(37)
Mϕ-EVs	Exposed or not to either LPS or to stationary phase *Leishmania mexicana* promastigotes	Parasite infection (*Leishmania)*	Mexicana surface protease GP63	Induce signaling molecules and transcription factors in naive macrophages	(38)
Mϕ-EVs	Exposed to calcium oxalate monohydrate crystals	Kidney stone disease	L-plastin, coronin-like protein, pyruvate kinase, actin-related protein 3, HSP90β, and vimentin	Activate inflammasome, promote monocyte and T-cell migration, monocyte activation and macrophage phagocytic activity	(39)
Mϕ-EVs	–	Inflammation brain	Brain derived neurotrophic factor	Interact with brain microvessel endothelial cells *via* the integrin LFA-1 and ICAM-1, the carbohydrate-binding C-type lectin receptors	(40)
Mϕ-EVs	Stimulated with angiotensin II	Hypertension	ICAM-1 and PAI-1, miR-17	Increase the expression of ICAM1 and PAI-1 in human coronary artery endothelial cells	(41)
Mϕ-EVs	Mock-infected or infected with the macrophage-tropic HIV-1 BaL strain	HIV	48 miRNAs (e.g., miR-29a, miR-150)	Unclear	(42)
M2a-EVs, M2b-EVs, M2c-EVs	–	–	MRNA of Il1b、CCL2、CCL7、CCL3, Pf4	Affect the TLR, TNF, NLR, and NF-κB signaling pathways in recipient cells	(43)
M2-EVs	–	Lung cancer	AGAP2-AS1	Strengthen the radioresistance of radioresistant lung cancer cells *via* upregulating NOTCH2 and downregulating miR-296	(44)
M2-EVs	–	Pancreatic cancer	LncRNA SBF2-AS1	Suppress tumorigenic ability of pancreatic cancer *via* repressing miR-122-5p and upregulating XIAP	(45)
M2-EVs	–	Hypertrophic scar	LncRNA-ASLNCS5088	Modulate glutaminases expression in fibroblasts *via* targeting miR-200c-3p	(46)
M1-EVs	–	Inflammatory bowel disease	MiR-21a-5p	Decrease E-cadherin expression and excessively activate ILC2 *via* promoting GATA-3	(47)
M1-EVs	–	Myocardial infarction	MiR-155	Suppress Sirt1/AMPKα2-endothelial nitric oxide synthase and RAC1-PAK2 signaling pathways through targeting RAC1, PAK2, Sirt1, and AMPKα2	(48)
M2-EVs	Treated with IL-4-	AS	MiR-99a/146b/378a	Target NF-κB and TNF-α signaling pathways to suppress inflammation	(49)
Mϕ-EVs	–	Idiopathic pulmonary fibrosis	MiR-142-3p	Decrease the expression of TGFβ-R1 and profibrotic genes in alveolar epithelial cells and lung fibroblasts	(50)
Mϕ-EVs	Induced by LPS	Inflammation	MiR-146a、miR-146b, miR -21-3p	Secrete various chemokines and cytokines, activate Immune signaling pathways	(51)
Mϕ-EVs	Induced by nicotine	AS	MiR-21-3p	Promote vascular smooth muscle cells proliferation and migration through targeting PTEN	(52)
Mϕ-EVs	Stimulated by oxidized low-density lipoprotein	AS	MiR-146a	Increase the release of reactive oxygen species ROS and neutrophil extracellular traps NETs *via* targeting SOD2	(53)
Mϕ-EVs	Induced by deoxycholic acid	Intestinal metaplasia	MiR-30a-5p	Promote the CDX2 expression and suppressed the proliferation of human gastric epithelial cells by targeting FOXD1	(54)
Mϕ-EVs	Induced by LPS	Hepatic fibrosis	MiR-103-3p	Target KLF4 to promote the proliferation and activation of hepatic stellate cells	(55)
Mϕ-EVs	Stimulated by Treponema pallidum	Syphilis	MiR-146a-5p	Suppress monocyte transendothelial migration and endothelial permeability *via* targeting JAM-C	(56)
M2-EVs	–	Fracture	MiR-5106	Induce bone mesenchymal stem cells towards osteoblastic fate by targeting salt-inducible kinase 2 and 3	(57)
Diabetic-Mϕ-EVs	–	Diabetic fracture	MiR-144-5p	Inhibit bone mesenchymal stem cells osteogenesis differentiation by targeting Smad1	(58)
Mϕ-EVs	High glucose–treated	Type 2 diabetes	MiR-210	Bind with mRNA sequences of NDUFA4 gene to impair glucose uptake and mitochondrial complex IV activity	(59)
Mϕ-EVs	–	Spontaneous abortion	MiR-153-3p	Suppress the proliferation and migration of trophoblast cells through the IDO/STAT3 pathway.	(60)
M2-EVs	–	Pulmonary fibrosis	MiR-328	Enhance pulmonary interstitial fibroblast proliferation by targeting FAM13A	(61)
M1-EVs	Hypoxia/serum deprivation-induced	Myocardial infarction	MiR-222	Promote BMSCs apoptosis by targeting Bcl-2	(62)
Mϕ-EVs	–	Ischemia-reperfusion injury	MiR-148a	Suppress the expression of thioredoxin-interacting protein and inactivate the TLR4/NF-κB/NLRP3 signaling pathway	(63)
Mϕ-EVs	Stimulated by hypoxia-reoxygenation	Ischemia-reperfusion injury	MiR-29a	Promote inflammatory cytokines secretion and cardiomyocyte pyroptosis by targeting MCL-1	(64)
Mϕ-EVs	–	Type 2 diabetes	MiR-29a	Induce insulin resistance through targeting PPARγ signaling	(65)
M1-EVs	–	Carotid artery injuries	MiR-222	Target CDKN1B and CDKN1C to promote vascular smooth muscle cell proliferation and migration	(66)
M2-EVs	–	Acute myocardial infarction	MiR-1271-5p	Decrease cardiomyocyte apoptosis *via* decreasing SOX6 expression	(67)
Mϕ-EVs	Activated by Toll-like receptor 3	Hepatitis C virus infection	MiR-29	Induce the expression of IFN-α- and IFN-stimulated genes (ISGs, MxA, OAS-1, and OAS-2) in human hepatic cells	(68)
M1-EVs	–	Breast cancer	MiR-130, MiR-33	Perform anti-tumor effect by polarizing Mϕ from M2 to M1 phenotype	(69)
M2-EVs	–	Asthma	MiR-370	Reduce cell apoptosis, relive inflammation *in vitro* and *in vivo* through suppressing the FGF1/MAPK/STAT1 axis	(70)

MMP-9, Matrix metalloproteinase 9; NLRP3, Nod-like receptor protein 3; NF-κB, Nuclear factor kappa-light-chain-enhancer of activated B cells; TGF-β, Transform growth factor-β; Smad2/3, Small mothers against decapentaplegic 2/3; MAPK, Mitogen-activated protein kinases; ER, Endoplasmic reticulum; HUVECs, Human umbilical vein endothelial cells; LFA-1, Lymphocyte function-associated antigen 1; ICAM-1, Intercellular adhesion molecule 1; PAI-1, Plasminogen activator inhibitor-1; TLR, Toll-like receptors; TNF, Tumor necrosis factor; NLR, NOD-like receptor; RAC1, RAS-related C3 botulinus toxin substrate 1; PAK2, p21-activated kinase 2; Sirt1, Sirtuin 1; AMPKα2, Adenosine monophosphate-activated protein kinas alpha 2; AS, Atherosclerosis; PTEN, Phosphatase and tensin homolog; ROS, Reactive oxygen species; NETs, Neutrophil extracellular traps; SOD2, Superoxide dismutase 2; CDX2, Caudal-related homeobox transcription factor 2; FOXD1, Forkhead Box D1; KLF4, Krüppel-like factor 4; JAM-C, Junctional adhesion molecule C; NDUFA4, NADH dehydrogenase ubiquinone 1 alpha subcomplex 4; IDO, Indoleamine 2,3-dioxygenase; STAT3, Signal Transducers and Activators of Transcription 3; FAM13A, Family with sequence similarity 13, member A; Bcl-2, B-cell lymphoma -2; MCL-1, Myeloid cell leukemia-1; PPARγ, Peroxisome proliferator-activated receptor gamma; CDKN1B, Cyclin Dependent Kinase Inhibitor 1B; CDKN1C, Cyclin Dependent Kinase Inhibitor 1C; SOX6, Sox family transcription factors 6; FGF1, Fibroblast growth factor 1.

However, there is no consensus on the specific markers of EVs subtypes according to the *MISEV2018* (23). In this review, we primarily focus on EVs 150 nm or less in size.

### Molecular Components of Mφ-EVs

The RNA molecules enclosed in Mφ-EVs comprise mainly mRNA (intact mRNA and mRNA fragments) (31, 43), miRNA (60), long non-coding RNA (46), and tRNA (1). Lee et al. obtained EVs from Mφ treated with Shiga toxin 2a toxoids and found that they express higher levels of mRNAs encoding the pro-inflammatory cytokines IL-1β and IL-8, thereby exacerbating inflammation (9). Zhu et al. evaluated the features of Mφ-EVs and EVs derived from tumor-associated Mφ (TAM-EVs) (72) and found that different RNA processing proteins resulted in different RNA profiles. These results indicate that EVs that transport mRNA may be internalized and translated. MiRNA incorporated in EVs could circulate in the blood without degradation from blood RNAse activity (3). In addition, Mφ-EV miRNAs participate in the immune response (51), induce mesenchymal stem cell differentiation (57), regulate the tumor-associated microenvironment (73, 74), and mediate cell proliferation and migration (52). However, to date, few studies have investigated the presence of DNA in EVs (3).

In recent years, proteomic studies have provided new insights into the protein components of Mφ-EVs. Yao et al. reported a number of differentially expressed proteins in IFN-α-treated Mφ-EVs (35), including 74 upregulated and 20 downregulated proteins involved in antiviral-related pathways. In another study, 22 upregulated proteins in LPS-induced Mφ-EVs activated the NOD-like receptor signaling pathway and the NLRP3 inflammasome in patients with acute liver injury (25). Huang et al. screened proteins from silica-exposed Mφ-EVs and identified 291 differentially expressed proteins; the SPP1 protein was found to play a critical role in the response to silicosis (36). These findings emphasize that the biological functions of EV proteins vary under different conditions.

EVs can also deliver soluble mediators such as cytokines and enzymes. Haque et al. investigated the role of Mφ-EVs exposed to cigarette smoke condensate in HIV patients and found an association with catalase upregulation (29). In colitis patients, EVs transmitting CC chemokine 1 directly interact with CCR8 to alleviate colon damage and relieve inflammation (12).

Lipids also play an important role in the functions of Mφ-EVs. Kadiu et al. applied lipidomic analysis to explore how Mφ-EVs facilitate HIV-1 infection (75) and found that MVs and exosomes derived from Mφ have unique lipid profiles. Specifically, viral membranes enriched with lipids, such as glycerophosphoserine, sphingomyelin, and dihydrosphingomyelin, were readily detected in the MV fraction; by contrast, phosphatidylethanolamine/ceramide was identified only in the exosome population (75). However, the complete lipid profiles of Mφ-EVs are poorly understood and require further study.

### Biogenesis of Mφ-EVs

The mechanisms of microvesicle biogenesis are associated with outward budding and fission of the plasma membrane. Apoptotic bodies are released as blebs in cells undergoing apoptosis (3). The biogenesis of exosomes is a complex and dynamic process. Invagination of the plasma membrane initiates the first step of exosome biogenesis and produces endocytic vesicles. The fusion of multiple endocytic vesicles leads to the formation of early endosomes (EEs). Many intercellular cargoes are encapsulated in such EEs in a clathrin- or caveolin-dependent or independent manner (76, 77). With the assistance of the Golgi complex, EEs invaginate and mature into late endosomes known as multivesicular bodies (MVBs) (2). Inward membrane budding results in the formation of intraluminal vesicles (ILVs), which are housed in MVBs (78). MVBs show two types of reversion: they may fuse with the plasma membrane and release ILVs into the extracellular space as exosomes, or they may fuse with lysosomes or autophagosomes and ultimately lead to degradation (4). Endosomal sorting complex required for transport (ESCRT) is the best-described mechanism underlying MVB formation and protein sorting in MVBs (79). ESCRT includes four different protein complexes, ESCRT-0, -I, -II, -III (80). The ESCRT-0, -I and ESCRT-II complexes form a recognition domain in the endosomal membrane that recognizes and ubiquitinates membrane proteins. The ESCRT-III complex is responsible for membrane budding and the release of ILVs (81). Other critical players include Sytenin-1, TSG101, ALIX, Rab GTPases, Pmel17, and tetraspanins (4). In addition to proteins and ceramides, lipids such as sphingomyelinases are also involved in the biogenesis of exosomes (82).

Furthermore, stimulation could influence Mφ-EV cargo sorting or ultimate release in the biogenesis of Mφ-EVs. Mφ exposed to irradiated apoptotic cancer cells activate peroxisome proliferator-activated receptor gamma (PPARγ) and increase the expression of phosphatase and tensin homolog (PTEN) in Mφ-EVs (83). Similarly, Mφ exposed to extracellular adenosine triphosphate (ATP) activate the P2X7 signaling pathway and increase calpain activity, ultimately leading to IL-1β expression and loading of unconventional proteins into Mφ-EVs (84, 85). Interestingly, the more released of Mφ-EVs would result from lipopolysaccharide (LPS) stimulation, and the mechanism is related to upregulation of Rab27a and Rab27b, while it is inhibited by IL-25 (86).

### Relationship Between Mφ and Mφ-EVs

The description of the Mφ phenotype is widely accepted: classically activated or inflammatory M1Mφ are induced by IFN-γ, TNF-α or bacterial LPS, whereas alternatively activated or anti-inflammatory M2Mφ are polarized by IL-4 and IL-13 (87, 88). Plasticity is an important property of Mφ (89). Cytokines in the microenvironment can alter the phenotype of Mφ (90). Different phenotypes have different functions; for example, M1Mφ secrete higher levels of pro-inflammatory cytokines, exerting potent antimicrobial and antitumor activities that impair tissue regeneration and wound healing (91–93). By contrast, M2Mφ have anti-inflammatory effects that remove debris and apoptotic cells, promote angiogenesis, and facilitate fibrosis, tissue repair, and wound healing (94–96).

There are three major phenotypes of Mφ-EVs: unpolarized M0Mφ-derived EVs (M0-EVs), M1Mφ-derived EVs (M1-EVs), and M2Mφ-derived EVs (M2-EVs) (97). Their biological functions vary depending on the parental cell properties. For instance, in the pathogenesis of atherosclerosis (AS), M1-EVs containing a high level of miR-155 suppress the proliferation of fibroblasts and promote the development of AS (98), while M2-EVs deliver miR-1271-5p to suppress cardiomyocyte apoptosis and perform cardiac repair (67). Moradi-Chaleshtori et al. reported that M1-EVs polarize Mφ from the M2 to M1 phenotype and have antitumor effects by carrying miR-130 and miR-33 (69). M2-EVs promote cell invasion in breast cancer by transporting miR-223 to target the Mef2c/β-catenin pathway (99). Furthermore, each EV phenotype has polarization-specific control in the bone repair process. M0-EVs and M2-EVs enhance bone regeneration, while M1-EVs inhibit bone repair (100).

## Immunomodulatory Effects of Mφ-EVs in Chronic Inflammatory Disease

Inflammation is the body’s defense response to stimuli such as infection or injury, and it can be divided into acute and chronic phases. Acute inflammation is rapid in onset and short in duration, mainly characterized by exudative lesions. Chronic inflammation can lead to pathological changes in tissues and organs, which in turn cause diverse chronic diseases, including diabetes, cancer, cardiovascular disease, respiratory disease, and gastrointestinal disease (101, 102).

Chronic diseases are driven by pathological inflammation, eventually leading to tissue damage (103). In short, disorders resulting from inflammation-related pathways are the primary mechanism leading to chronic disease.

**Figure 2:**
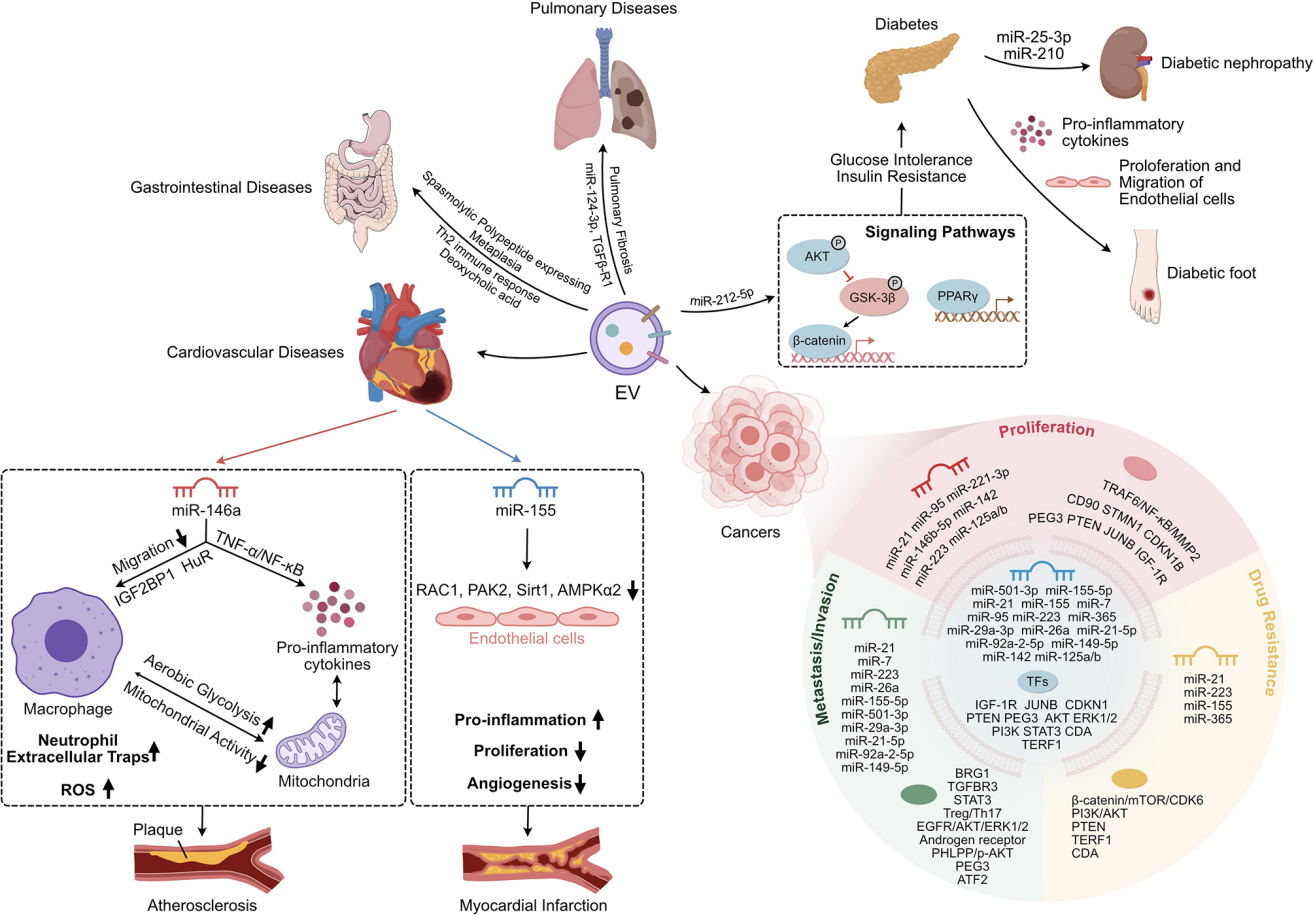
The Immuno-modulation Effect of Mφ-EVs in chronic inflammatory diseases. Mφ-EVs regulate the immune response and cell proliferation and migration and are involved in signaling pathways in the development of chronic inflammatory disease. Mφ-EVs, Macrophage-derived extracellular vesicles; Th2, CD4^+^ T helper 2 lymphocytes; AKT, Protein kinase B; GSK-3β, Glycogen synthase kinase-3beta; PPARγ, Peroxisome proliferator-activated receptor gamma; TNF-α, Tumor necrosis Factor alpha; NF-κB, Nuclear factor kappa-light-chain-enhancer of activated B cells; IGF2BP1, Insulin-like growth factor 2 mRNA-binding protein 1; HuR, Human antigen R; ROS, Reactive oxygen species; RAC1, RAS-related C3 botulinus toxin substrate 1; PAK2, p21-activated kinase 2; Sirt1, Sirtuin 1; AMPKα2, Adenosine monophosphate-activated protein kinas alpha 2; TRAF6, TNF receptor associated factor 6; MMP2, Matrix metalloproteinase 2; CD90, Cluster of differentiation 90; STMN1, Stathmin 1; CDKN1B, Cyclin-dependent kinase inhibitor 1B; PEG3, Paternally expressed gene 3; PTEN, Phosphatase and tensin homolog; JUNB, AP-1 transcription factor; IGF-1R, Insulin-like growth factor receptor; mTOR, Mammalian target of rapamycin; CDK6, Cyclin-dependent kinase 6; PI3K, Phosphatidylinositol-3-kinase; TERF1, Telomeric repeat binding factor 1; CDA, Cytidine deaminase; BRG1, Brahma-related gene 1; TGFBR3, TGF-beta type III receptor; STAT3, Signal Transducers and Activators of Transcription 3; Treg, regulatory T lymphocytes; Th17, IL-17-producing CD4^+^ T lymphocytes; EGFR, Epidermal growth factor receptor; ERK1/2, Extracellular signal-regulated kinase 1/2; PHLPP, PH domain leucine-rich-protein phosphatase; p-AKT, Phosphorylated-Akt; ATF2, Activating transcription factor 2.

EVs play a vital role in maintaining tissue homeostasis and regulating disease progression as another mode of cellular interaction (104, 105). Numerous studies have elucidated the impact of Mφ-EVs on chronic inflammation and disease. EVs derived from different Mφ have unique effects under various pathological conditions (106, 107) (**Figure 2**).

### Cardiovascular Disease

AS plays a prominent role in coronary heart disease, cerebral infarction, and peripheral vascular disease (108). It is a multifactorial disease, and its exact pathogenesis has not been elucidated. Hypertension, hyperlipidemia, obesity, smoking, and diabetes are all risk factors for its development (109). AS is characterized by lesions in affected arteries starting from the intima, with lesions involving mainly large and medium arteries. It can lead to deposits of lipids and complex sugars, hemorrhage, thrombus formation, and many other conditions that eventually thicken and stiffen the arterial wall and narrow the vessel lumen. AS plaques are composed primarily of immune, foam, and inflamed smooth muscle cells (110).

AS is an inflammatory response caused by the retention of cholesterol-rich, B-type lipoproteins in susceptible areas of medium and large arteries (111). Dysregulation of the balance between cellular and systemic cholesterol promotes the deposition of such lipoproteins in the arterial wall (109).

The development of AS is closely associated with endothelial cell damage, vascular inflammation, and massive accumulation of Mφ. The imbalance between Mφ recruited to plaques and Mφ migration from plaques to regional lymph nodes causes deposition of lipid-laden Mφ in the arterial wall, further promoting the inflammatory progression of AS. Thus, Mφ migration regulates the development of AS (108, 112). EVs mediate intercellular communication in atherosclerotic plaques (113). Furthermore, activated monocytes or Mφ-EVs can propagate inflammatory signals and modulate AS development *via* various pathways (114–116). Nguyen et al. reported that EVs secreted from Mφ loaded with oxidized low density lipoprotein are taken up by naive Mφ and inhibit Mφ migration *in vitro* and *in vivo* (116). In addition, Mφ-EVs can block the migration of Mφ to the chemokine stimulator CCL2, and EVs transport miR-146a, which suppresses the expression of genes in Mφ related to cell migration, such as HuR and IGF2BP1 (116). Mφ may also promote the progression of AS *via* activation of the NF-κB pathway. The modulatory effect of Mφ-EVs on AS is further evidenced by their ability to regulate hematopoietic stem cells differentiation and necrosis by suppressing the TNF-α/NF-κB signaling pathway (49). Furthermore, Zhu et al. isolated EVs from nicotine-treated Mφ and illustrated that they aggravate the development of AS by transporting the exosomal miR-21-3p target PTEN, which enhances vascular smooth muscle cell migration and proliferation (52).

Oxidative stress and inflammation are closely related, and they form a feed-forward loop that promotes the development of AS. During AS, Mφ produce significant levels of reactive oxygen species (ROS) *via* mitochondrial metabolism (109). M1Mφ enhance aerobic glycolysis and reduce mitochondrial activity. In contrast, M2Mφ mediate mitochondrial oxidative phosphorylation (117). Bouchareychas et al. isolated EVs from mouse bone marrow-derived Mφ treated with IL-4 and applied these EVs to naive BMDMs, which resulted in enhanced ATP production and cellular respiration. These results indicate that EVs can effectively regulate cellular reprogramming by improving energy metabolism (49). Mφ-derived EVs treated with superoxide dismutase 2, a target of oxidized low density lipoprotein, showed increased ROS production and the release of neutrophil extracellular traps *via* miR-146a (53).

Mφ modulation plays an essential role in immunomodulatory processes during cardiac repair and in remodeling post-myocardial infarction (118). Hypoxia/serum deprivation-induced M1-EVs transport miR-222, which promotes apoptosis and inhibits viability in bone marrow mesenchymal stem cells by targeting B-cell lymphoma-2 (62). miR-155 is predominantly expressed in Mφ and cardiac fibroblasts and is one of the most abundant miRNAs in M1-EVs (119). The expression of miR-155 is upregulated in Mφ-EVs from the hearts of mice after acute myocardial infarction (AMI). EVs enriched with miR-155 inhibit the proliferation of fibroblasts and promote the inflammatory response; a deficiency of miR-155 decreases the incidence of cardiac rupture after AMI and improves cardiac function (98). Previous studies have observed angiogenesis-inhibiting effects of M1Mφ, but the mechanism of action is not entirely understood (120). M1-EVs transfer miR-155 to endothelial cells, reducing their angiogenic ability by downregulating miR-155 target genes including RAC1, PAK2, Sirt1, and AMPKα2 (48). Thus, inhibition of miR-155 expression may be a novel method for the clinical treatment of MI. By contrast, M2-EVs transport miR-1271-5p, which suppresses apoptosis in cardiomyocytes and performs a cardiac repair function in AMI by targeting SOX6 (67).

Generally, ischemia–reperfusion is applied to restore coronary artery blood flow and relieve disease progression. However, numerous studies have demonstrated that ischemia–reperfusion injury (IRI) can lead to cardiac dysfunction due to calcium overload and overproduction of free radicals such as ROS (121). Induction of hypoxia–reoxygenation is the main method for establishing IRI in animal models (64). Wang et al. demonstrated that Mφ subjected to hypoxia–reoxygenation are polarized toward M1Mφ and derived EV miR-29a to promote cardiomyocyte pyroptosis by targeting myeloid cell leukemia-1 (64). M2-EVs alleviate cardiac dysregulation and Ca^2+^ overload, which relieves IRI. miR-148a within M2-EVs suppresses the expression of thioredoxin-interacting protein and inactivates the TLR4/NF-κB/NLRP3 signaling pathway (63).

### Diabetes Mellitus

The incidence of type 2 diabetes has risen sharply over the past few decades, with a global prevalence of over 300 million people (122). Type 2 diabetes accounts for approximately 95% of diabetes cases and is characterized by hyperglycemia due to insulin resistance (IR) and relative insulin deficiency (123, 124). Obesity is a risk factor for type 2 diabetes. Moreover, chronic low-grade inflammation is a leading cause of obesity-induced IR (125).

The massive accumulation of pro-inflammatory Mφ in adipose tissue and the liver is a distinguishing feature of obesity-induced chronic inflammation in tissues (126). Mφ could be the ultimate effector cells that secrete the major cytokines responsible for IR. Mφ in normal adipose tissue express CD206 receptors and release Arg-1, but those in inflamed tissues are polarized toward the M1 phenotype (127). In a previous study, administering EVs derived from adipose tissue Mφ (ATM-EVs) from obese mice to lean mice caused glucose intolerance and IR, while administering lean mice ATM-EVs to obese mice improved glucose tolerance and insulin sensitivity (128).

Chronic low-grade tissue inflammation is the main cause of IR, leading to islet β cell failure. Qian et al. analyzed the effects on β cells of M1-EVs and EVs isolated from islet-resident Mφ from mice fed a high-fat diet (129) and found that miR-212-5p restricted insulin secretion by targeting the SIRT2 gene and regulating the Akt/GSK-3β/β-catenin pathway (129). Moreover, miR-155, which is overexpressed in obese ATM-EVs, suppresses the expression of PPARγ and impairs the inhibitory effects of insulin on glucose production (128). Similarly, miR-29a within ATM-EVs promotes obesity-induced IR by directly targeting PPARγ (65).

Many complications can occur during the late stages of diabetes. The persistence of diabetic inflammation activates inflammatory cells, which secrete inflammation-associated cytokines (130). A common complication of diabetes is difficulty healing. Mφ-EVs show a remarkable decrease in the release of pro-inflammatory cytokines, enhancing the proliferation and migration of endothelial cells and accelerating wound healing *via* their anti-inflammatory effects (131). Similar research has shown that M1-EVs can regulate Mφ phenotypic reprogramming, repolarizing M1Mφ to M2Mφ, which in turn promotes wound healing (132). Bone homeostasis is also disturbed by diabetes mellitus. Zhang et al. isolated EVs from diabetic bone marrow-derived Mφ and found that they transport miR-144-5p, which inhibits osteogenesis differentiation by targeting Smad1 and suppressing facture repair *in vivo* (58). Diabetic nephropathy is a peripheral small artery occlusive disease caused by diabetic neuropathy and lower extremity vasculopathy. M2-EVs that transport miR-25-3p may alleviate podocyte injury induced by high glucose levels by activating autophagy of the cells through suppression of DUSP1 expression (133). Zhu et al. found that Mφ-derived EVs treated with high levels of glucose might activate glomerular mesangial cells through the TGF-β1/Smad3 pathway, promote proliferation, and induce the production of fibrotic and inflammatory factors (31). Similarly, high-glucose-treated Mφ-derived EVs induce overexpression of inflammatory cytokines and activate NF-κB p65 signaling pathways (134). ATM-EVs contain high levels of miR-210, and miR-210 in ATM-EVs has been shown to regulate glucose uptake and mitochondrial chain complex IV activity by targeting NDUFA4 gene expression, which promotes the pathogenesis of diabetes (59).

These findings highlight the importance of Mφ-EVs in adipose tissue and suggest that the contents and functions of ATM-EVs vary with the ATM phenotype.

### Cancer

Cancer is the leading cause of death worldwide (135). In 2012, approximately 14.1 million new cancer cases and 8.2 million cancer deaths were recorded worldwide (135). Furthermore, with the aging population, the incidence and mortality rate of cancer have rapidly been increasing, imposing a significant burden on society. Inflammation plays a crucial role in the development of cancer (136). The “seed and soil” theory proposed in 1889 (137) compares cancer cells to seeds and the human microenvironment to soil and postulates that whether a tumor metastasizes depends on whether the soil meets the growth conditions of the seed (137). In recent years, more studies have focused on the regulatory roles of active immune cells such as Mφ, neutrophils, and mast cells in the tumor microenvironment (TME) (138, 139). Among such cells, TAMs play the most critical role in the TME (140, 141).

Mφ-EVs have diverse effects on the TME under various pathological conditions (4, 142, 143). For example, EVs transport apolipoproteins from TAMs to gastric cancer cells to promote cell migration (144), the expression of matrix metalloproteinase-2, and the pathogenesis of abdominal aortic aneurysms by activating the JNK and p38 signaling pathways (145).

EVs mediate intercellular communication in the TME *via* miRNA-induced epigenetic modifications in recipient cells. EVs miRNAs can regulate tumor cell migration, invasion, and drug resistance *via* various mechanisms, which in turn affect tumorigenesis (**Table 2**).

**Table 2:** The biological functions of Mφ-EV miRNAs in TME.

EVs sources	Disease model	MiRNAs	Mechanism	Reference
M1-EVs	Breast cancer cells	MiR-130, MiR-33	Perform anti-tumor effect by polarizing macrophage from M2 to M1 phenotype	(69)
M2-EVs	Colorectal cancer	MiR-21-5p, MiR-155-5p	Downregulate BRG1 expression, enhance colorectal cancer cells migration and invasion	(107)
M2-EVs	GC	MiR-21	Suppress cell apoptosis and strengthen activation of PI3K/AKT signaling pathway *via* down-regulation of PTEN	(146)
TAMs-EVs	PDAC	MiR-501-3p	Promote the PDAC cells invasion, migration and tube formation through the downregulation by activating the TGF-β signaling pathway to downregulate TGFBR3	(147)
TAMs-EVs	Prostate cancer	MiR‐95	Downregulate the downstream gene, JunB, to promote PCa cell proliferation, invasion, and epithelial-mesenchymal transition	(148)
M2-EVs	EOC	MiR-221-3p	Suppress CDKN1B to enhance the proliferation and G1/S transition of EOC cells	(73)
Mϕ-EVs	EOC	MiR-223	Induce cell drug resistance by activating PTEN-PI3K/AKT pathway	(149)
TWEAK-stimulated macrophages-EVs	EOC	MiR-7	Inhibition of tumor metastasis and aggressiveness *in vitro* and *in vivo via* EGFR/AKT/ERK1/2 signaling pathway	(150)
TAMs-EVs	EOC	MiR-146b-5p	Inhibit the HUVECs migration by activating TRAF6/NF-κB/MMP2 pathway.	(151)
TAMs-EVs	EOC	MiR-29a-3p, MiR-21-5p	Suppress STAT3 expression and regulate the ratio of Treg/Th17	(152)
M2-EVs	Breast cancer cells	MiR-223	Target the Mef2c-β-catenin pathway and promote breast cancer cell invasion	(99)
Mϕ-EVs	HCC	MiR-142, MiR-223	Inhibit HCC proliferation through suppressing STMN1 and IGF-1R expression	(153)
TAMs-EVs	HCC	MiR-125a/b	Suppress cell proliferation and stem cell properties by targeting CD90	(154)
Mϕ-EVs	HCC	MiR-92a-2-5p	Suppress androgen receptor translation, modify the PHLPP/p-AKT/β-catenin signaling to increase liver cancer cells invasion	(74)
M2-EVs	HCC	MiR-149-5p	Promote the invasion and migration of HCC by increasing MMP9 pathway	(155)
TAMs-EVs	PDAC	MiR-365	Upregulate pyrimidine metabolism and increase NTP levels in cancer cells, upregulating CDA to promote gemcitabine resistance	(156)
TAMs-EVs	Neuroblastoma cells	MiR-155	Downregulate TERF1 expression to increase CDDP resistance both *in vitro* and *in vivo*	(157)
M2-EVs	Glioma cells	MiR-21	Promote migration, proliferation and invasion, suppress apoptosis of glioma cells by reducing PEG3 expression	(158)
M2-EVs	Bladder carcinogenesis	MiR-21	Promote cell migration and induce cell CDDP resistance	(159)
M2-EVs	Esophageal Cancer	MiR-26a	Regulate the impacts of overexpressed AFAP1-AS1 on cell migration and invasion	(160)

GC, Gastric cancer; PDAC, Pancreatic ductal adenocarcinoma; EOC, Epithelial ovarian cancers; HCC, Hepatocellular carcinoma; PI3K, Phosphatidylinositol-3-kinase; AKT, Protein kinase B; PTEN, Phosphatase and tensin homolog; TGFBR3, TGF-beta type III receptor; JUNB, AP-1 transcription factor; CDKN1B, Cyclin-dependent kinase inhibitor 1B; EGFR, Epidermal growth factor receptor; ERK1/2, Extracellular signal-regulated kinase 1/2; TRAF6, TNF receptor associated factor 6; MMP2, Matrix metalloproteinase 2; NF-κB, Nuclear factor kappa-light-chain-enhancer of activated B cells; STAT3, Signal Transducers and Activators of Transcription 3; Treg, regulatory T lymphocytes; Th17, IL-17-producing CD4^+^ T lymphocytes; STMN1, Stathmin 1; IGF-1R, Insulin-like growth factor receptor; CD90, Cluster of differentiation 90; PHLPP, PH domain leucine-rich-protein phosphatase; p-AKT, Phosphorylated-Akt; MMP9, Matrix metalloproteinase 9; CDA, Cytidine deaminase; CDDP, Cisplatin; PEG3, Paternally expressed gene 3; TERF1, Telomeric repeat binding factor 1; Mef2c, Myocyte enhancer factor; AFAP1-AS1, Actin filament associated protein 1 antisense RNA1.

In recent years, proteomics has dramatically facilitated the study of proteomic profiles of EVs, and research on Mφ-EVs has provided new insights into how the TME is regulated (161, 162). A thorough comparative proteomic analysis of EVs revealed that TAM-derived exosomal proteins are responsible mainly for RNA processing and proteolytic functions and determined that recipient cells have an improved capacity to degrade denatured or misfolded proteins after uptake of TAM-EVs, which enhances their survival in the TME (72). Furthermore, TAM-EVs have molecular profiles associated with Th1/M1 polarization profiles; they enhance inflammation and immune responses and promote the proliferation and activation of T cells *ex vivo*. Thus, TAM-EVs are potent stimulators of antitumor immunity (163). They also activate the matrix metalloproteinase‐9 signaling pathway to promote hepatocellular carcinoma tumor migration by mediating the intercellular transfer of αMβ2 (CD11b/CD18) (24).

Recently, it was found that EVs contain damaged DNA from the nucleus and mitochondria, regulating tumor immunity *via* paracrine and activated cytoplasmic DNA sensor pathways and in specific immune cell subpopulations (164). After chemotherapy, exposing Mφ to apoptotic breast cancer cells causes higher levels of IL-6 to be released and delivered to cancer cells *via* increased phosphorylation of STAT3, promoting the proliferation and metastasis of the cells (8). Furthermore, Mφ-EVs may synthesize proteins such as thromboxane and thromboxane B2 (163). However, it is unclear whether they contain functional endogenous DNA. In oral squamous cell carcinoma, THP-1-derived EVs and native human Mφ-derived EVs have been shown to activate the AKT/GSK-3β signaling pathway, reducing the proliferative effects of 5-FU and cis−diamminedichloroplatinum (CDDP) and the apoptosis of OSC-4 cells (165).

Metabolic reprogramming, an essential hallmark of malignancy, is regulated by the microenvironment. TAM-derived EVs enhance aerobic metastasis and the anti-apoptotic ability of carcinoma cells by transporting a myeloid-specific long non-coding RNA and HIF-1α-stabilizing long non-coding RNA (HISLA) (166). HILSA inhibits the hydroxylation and degradation of HIF-1α, whereas carboxylic acid secreted from growing glycolytic cells upregulates HISLA in TAMs, constituting a feed-forward loop between TAMs and growing cells. Thus, HISLA inhibits metastasis and chemoresistance in carcinoma *in vivo* (166). This suggests that EVs-mediated metabolic reprogramming plays an important role in the intercellular communication between immune and tumor cells. Azambuja et al. proposed that glioblastoma-derived EVs reprogram M1Mφ into TAMs and promote the pro-tumor functions of M2Mφ, while these GEV-reprogrammed TAM-EVs promote glioblastoma cell migration and proliferation (27).

In summary, because Mφ are sensitive to microenvironmental stimuli, the composition of their secreted EVs in different disease models change as the Mφ themselves are altered.

### Pulmonary Disease

Idiopathic pulmonary fibrosis (IPF) is an intermittent and chronic fibrotic lung disease associated with inflammatory immune damage, caused primarily by chronic alveolar epithelial injury and dysregulated wound healing due to abnormal proliferation of fibroblasts (167). The expression of miR-142-3p is markedly increased in the sputum and plasma of IPF patients, and Mφ-EVs reduce the expression of TGFβ receptor 1 by transporting miR-142-3p, suppressing the progression of pulmonary fibrosis (50). Antifibrotic miRNA delivery in the lung can effectively prevent pulmonary fibrosis and provide new therapeutic avenues for the treatment of IPF. In addition, M2-EVs may transport miR-328, facilitating the proliferation of pulmonary interstitial fibroblasts *via* family with sequence similarity 13, member A (61).

Another pulmonary fibrosis-related disease is silicosis, which is caused by long-term inhalation of large amounts of free silica dust and is characterized by extensive nodular fibrosis of the lungs (168). EVs have been isolated from silica-exposed Mφ and found to induce the proliferation of myofibroblasts and fibroblasts and increase their expression levels of SPP1 (36). They also induce the overproduction of proinflammatory cytokines and promote myofibroblast activation in an endoplasmic reticulum stress-dependent manner (30).

In acute lung injury, Mφ-EVs in alveolar lavage fluid release various pro-inflammatory factors mainly during the early stages of damage; this activates neutrophils to produce IL-10, which might be responsible for polarizing Mφ to M2c, leading to post-acute lung injury fibrosis (169).

Asthma, a chronic respiratory disease, is characterized by inflammation and hyperresponsiveness of the airways (170). M2-EVs deliver miR-370 to reduce cell apoptosis and relieve inflammation by suppressing the FGF1/MAPK/STAT1 axis (70). M2-EVs have also been found to upregulate AGAP2-AS1 and NOTCH2 expression and downregulate miR-296 expression to strengthen the radioresistance of lung cancer cells (44).

### Gastrointestinal Disease

Chronic inflammation influences the development of spasmolytic polypeptide-expressing metaplasia (171), and deoxycholic acid (DCA) enhances the expression of enteral metaplasia markers (172). Xu et al. cocultured mouse stomachic organoids with DCA treated macrophage-derived EVs(DCA-Mφ-EVs), and the data revealed that the expression of SPEM marker proteins TFF2, GSII, SPEM-related cistron Wfdc2, Olfm4 and Cftr were considerably inflated when 72h cocultured (172). In addition, miR-30a-5p enriched in Mφ-EVs derived from DCA promotes intestinal metaplasia and inhibits the proliferation of human gastric cancer cells by targeting forkhead box D1 (54). These results suggest that Mφ-EVs may mediate intercellular communication in the DCA microenvironment and promote the progression of spasmolytic polypeptide-expressing metaplasia, providing a new target for treating gastric intestinal metaplasia.

Chronic intestinal inflammation may eventually lead to inflammatory bowel disease (173). To assess the effects of Mφ-derived EVs on inflammatory bowel disease, Yang et al. isolated EVs from the M2aMφ, M2bMφ, and M2cMφ phenotypes and established a dextran sulfate sodium-induced colitis model in mice (12). Treating the mice with M2-EVs improved colon length. Furthermore, compared with EVs derived from M2aMφ and M2cMφ, M2bMφ-derived EVs were more effective. These EVs may interact with CCR8 by releasing the CCL1 chemokine, thereby increasing the expression of IL-4 and number of regulatory T cells to promote the Th2 immune response (12).

## Potential Therapeutic Strategies

EVs are considered a promising tool for immunotherapy, drug delivery, and targeted therapy. Furthermore, some strategies were proposed to strengthen the therapeutic capabilities and broaden the applications of EVs. Recent applications are discussed below (**Table 3**).

**Table 3:** The applications of Mφ-EVs.

EVs Source	Precondition of Macrophages	EVs Treatment	Disease model	Application	Reference
M2-EVs	–	–	Calvaria defects	Promote bone regeneration	(100)
Mϕ-EVs	Induced by LPS	–	Acute liver injury	Be involved in the activation of NLRP3 and NOD-like receptor signaling pathway	(25)
Mϕ-EVs	Induced by LPS	–	Ischemic stroke	Induce neuroprotection, and reduce the brain infarct	(174)
Mϕ-EVs	Induced by LPS	–	Chronic liver diseases	Promote hepatic stellate cells proliferation and activation	(55)
Mϕ-EVs	Treated with IL-4	–	AS	Reduce the areas of necrotic lesion	(49)
M2-EVs	–	–	Cutaneous wound	Promote wound healing	(132)
M1-EVs	–	–	Colorectal carcinoma	Enhance the anti-tumor effect of checkpoint inhibitors (anti-PD-L1 antibody) in cancer therapy	(175)
Mϕ-EVs	–	Loaded with Doxorubicin	Pancreatic cancer	Deliver Doxorubicin to perform anti-tumor efficacy	(176)
M1-EVs	–	Loaded with CDDP	Ovarian cancer	Increase cytotoxicity in drug-resistant by loaded with CDDP	(177)
Mϕ-EVs	*Infected by* M. bovis BCG	*-*	*M. tuberculosis* infection.	Induce a CD4^+^ and CD8^+^ memory T cell response and stimulate DC activation	(178)
M1-EVs	*-*	*-*	*Melanoma*	Enhance the efficacy of peptide vaccine, the cytotoxic T cell immune response and present anti-tumor effect	(179)
M2-EVs	–	–	Fracture	Induce bone mesenchymal stem cells osteogenic differentiation	(57)
Mϕ-EVs	–	–	Inflammation brain	Deliver the brain derived neurotrophic factor to the brain	(40)
Mϕ-EVs	–	Loaded with catalase	Parkinson’s disease	Deliver catalase to against oxidative stress, decrease brain inflammation and increase neuronal survival	(180)
Mϕ-EVs	–	Loaded with Edaravone	Stroke	Improve the bioavailability of Edaravone and strengthen the neuroprotective effects	(13)
Mϕ-EVs	–	Loaded with baicalin	Ischemic stroke	Improve the solubility of Baicalin, brain targeting ability and present neuroprotection	(181)
Mϕ-EVs	–	Loaded with PTX	Lung carcinoma	Deliver PTX to overcome multiple drug resistance and assess anti-cancer therapy	(182)
M2-EVs	–	Loaded with Berberine	Spinal cord injury	Deliver drugs to the injured spinal cord	(183)
Mϕ-EVs	–	Engeneered with AA-PEG vector moiety	Pulmonary metastases	Improve the loading capacity and therapeutic effects	(184)
M1-EVs	–	Modified with anti-CD47 and anti-SIRPα	Acidic tumor microenvironment,	Target tumors more effectively, reprogram M2Mφ to M1Mφ, exert anti-tumor function	(185)
Mϕ-EVs	–	Loaded with Biomimetic silibinin	Alzheimer’s disease	Inhibit astrocytes activation and alleviate astrocyte inflammation-mediated neuronal damage	(186)
Mϕ- A15 -EVs	Stimulated by phorbol 12-myristate 13-acetate	Loaded with Doxorubicin hydrochloride and co-incubated cholesterol-modified mi159	Triple-negative breast cancer	Co-deliver cholesterol-modified miRNA and chemotherapeutic drugs, perform more specific and robust targeting properties, and suppress tumor growth	(187)
Mϕ-EVs	–	Coated with poly (lactic-co-glycolic acid)	Triple-negative breast cancer	Improve the tumor-targeting, the cellular uptaking and the antitumor efficacy	(188)
M2-EVs	–	Modified with hexyl 5-aminolevulinate hydrochloride	AS	Enhance the anti-inflammatory effect and relieve AS	(189)
M1-EVs	–	Loaded with PTX	Breast cancer	Deliver PTX to enhance the anti-tumor activity	(190)

PTX, paclitaxel; AS, Atherosclerosis; AA-PEG, Aminoethylanisamide-polyethylene glycol; CDDP, Cisplatin; DC, Dendritic cells.

### Mφ-EVs as a Drug Candidate

As messengers carrying genes, proteins, and other biomolecules, EVs mediate communication in cells and have therapeutic functions. Kang et al. (25) applied M0-EVs, M1-EVs, and M2-EVs to rat calvaria defects; M2-EVs carrying miR-378a increased the expression of the mesenchymal stem cell osteoinductive genes BMP2 and BMP9 in the bone repair process to promote bone regeneration (100). Furthermore, proteomic profiling analysis of the protein composition of LPS-treated Mφ-EVs (L-Mφ-EVs) revealed that among 341 upregulated proteins in L-Mφ-EVs, 22 are involved in the NOD-like receptor signaling pathway. After L-Mφ-EVs were taken up by hepatocytes, NLRP3 was activated, which promoted acute liver injury (25). In ischemic stroke, microglia are converted into M1 phenotypes that release pro-inflammatory mediators, promoting neuronal apoptosis and brain injury (174). L-Mφ-EVs suppress inflammation, enhance microglial M2 polarization (which induces neuroprotection), and reduce the brain infarct volume *in vivo* after ischemic stroke (174). The miRNAs in Mφ-EVs are involved in different pathways to regulate the development of disease(Qian et al.; 7, 133). In chronic liver disease, L-Mφ-EVs have been shown to promote the proliferation and activation of hepatic stellate cells by enriching miR-103-3p and targeting Krüppel-like factor 4 (55). IL-4-treated Mφ-EVs transported anti-inflammatory miR-99a/146b/378a to inhibit inflammation by targeting NF-κB and TNF-α signaling, leading to delayed development of AS (49).

In addition, Mφ-EVs may have therapeutic functions *via* Mφ reprogramming. For example, M2-EVs promote wound healing by enhancing proliferation, angiogenesis, and collagen deposition (132), which suggests that Mφ phenotype reprogramming could provide valuable therapeutic options for the treatment of inflammation-related diseases. M1-EVs can also potentially be used to repolarize M2 to M1Mφ that secrete pro-inflammatory cytokines, have antitumor effects, and decrease tumor growth (175). Similarly, M1-EVs transport miR-130 and miR-33 to exert antitumor effects in breast cancer by polarizing Mφ from the M2 to M1 phenotype (69).

### Mφ-EVs as Drug-Delivery Systems

Many nanocarrier delivery systems have been designed to improve the efficacy of drugs, and EVs have the following advantages compared with other nanocarrier delivery systems: they transport a variety of endogenous biomolecules, and they are biocompatible, naturally targeted, and small enough to escape the clearance effect of the mononuclear phagocyte system (191, 192). Kanchanapally et al. (176) obtained EVs from different cells, including pancreatic cancer cells, pancreatic stellate cells, and Mφ loaded with doxorubicin (DOX), and compared their antitumor effects. Mφ-EVs loaded with DOX showed the highest antitumor efficiency followed by pancreatic stellate and pancreatic cancer cells. Zhang et al. isolated EVs from mononuclear M1Mφ and M2Mφ from umbilical cord blood and loaded them with CDDP; compared with the M2-EVs, M1-EVs showed increased cytotoxicity in drug-resistant A2780/DDP cells, suggesting that M1-EVs are a potential drug carrier in drug-resistant microenvironments (177).

The inability to cross the blood brain barrier (BBB) limits the application of 98% of therapeutic agents used for the treatment of CNS-related disorders (193). Recently, Yuan et al. (40) discovered that Mφ-EVs can cross the BBB and move into brain microvessel endothelial cells *via* integrin white corpuscle function-associated matter 1, living thing adhesion molecule 1, and carbohydrate-binding C-type glycoprotein receptors. That study further confirmed that intravenously injected Mφ-EVs crossed the BBB and transported brain-derived neurotrophic factor to the brain (40). A novel EVs-based formulation for catalase delivery in Parkinson’s disease patients was found to have neuroprotective effects against oxidative stress by inactivating ROS, decreasing brain inflammation, and increasing neuronal survival *in vivo* (180). That study provided a new theoretical basis for the development of other EVs-based drug-delivery systems for the treatment of CNS diseases, as well as an experimental basis for the in-depth study of the mechanisms involved in EV passage through the BBB. Silibinin, an antioxidant with poor brain targeting, has been applied to improve behavior and cognition in Parkinson’s patients. Huo et al. loaded Mφ-EVs with silibinin to improve its targeting capacity and released the silibinin to suppress astrocyte activation and relieve neuronal damage after crossing the BBB (186). Edaravone (Edv) delays neuronal death caused by acute cerebral infarction. Li et al. (13) prepared Mφ-EVs loaded with Edv, applied them to a rat model with permanent middle cerebral artery occlusion, and found that they notably improved bioavailability and prolonged the half-life of Edv. In addition, using these Edv-loaded Mφ-EVs, it was easier to target Edv to the ischemic side, and the treatment decreased neuronal death and promoted microglia M2 polarization *in vivo*.

Spinal cord injury severely damages the CNS. Gao et al. (183) developed an M2-EV-loaded berberine drug-delivery system that effectively prolonged the duration of berberine and improved its targeting capacity. In addition, it had anti-inflammatory and anti-apoptotic effects by repolarizing Mφ from the M1 to M2 phenotype. Mφ-EVs loaded with baicalin have been found to ameliorate the solubility and brain-targeting ability of baicalin, leading to significant neuroprotection in patients with ischemic stroke (181).

### Application of Engineered Mφ-EVs

Through genetic and chemical modifications, engineered EVs can enhance EVs targeting and therapeutic effects in cancer treatment (194). For example, Mφ loaded with paclitaxel show significant loading capacity, sustainable drug release, a profound capacity for accumulation in resistant cancer cells, and high cytotoxicity (182). Mφ loaded with paclitaxel and aminoethylbenzamide-polyethylene glycol readily accumulate in cancer cells and have a higher therapeutic effect *in vivo* compared with non-vectorized Mφ loaded with paclitaxel (184). M1-EVs tagged with anti-CD47 and anti-SIRPα using a pH-sensitive linker effectively target tumors, block SIRPα and CD47, and reprogram M2Mφ to M1Mφ, thereby exerting antitumor functions (185). Gong et al. stimulated THP-1 cells with phorbol 12-myristate 13-acetate to generate target-specific A15 EVs, packing Dox into them to codeliver cholesterol-modified miRNAs and chemotherapeutic drugs into triple-negative breast cancer cells; the system showed specific and robust targeting capabilities and suppressed tumor growth *in vivo* (187). To improve triple-negative breast cancer targetability, Li et al. (188) modified the surface of Mφ-EVs with a peptide to target mesenchymal–epithelial transition factor and developed a Mφ-EV-coated poly (lactic-co-glycolic acid) nanoplatform, which improved the efficiency of cellular uptake and the antitumor effects of DOX. Wu et al. (189) electroporated M2-EVs with FDA-approved hexyl 5-aminolevulinate hydrochloride, which produced anti-inflammatory carbon monoxide and bilirubin and further enhanced the anti-inflammatory effect by binding to surface-expressed chemokine receptors and releasing anti-inflammatory cytokines; they also relieved AS.

Modification of EVs may enhance their release of anticancer drugs and their antitumor effects by releasing pro-inflammatory Th1 cytokines. For example, M1-EV nano-formulation-loaded paclitaxel creates a pro-inflammatory environment that improves antitumor activity *via* the caspase-3 signaling pathway and exhibits antitumor effects *in vivo* (190).

In summary, Mφ-EVs have similar targeting and regulatory abilities as those of Mφ. Thus, the advantages of Mφ-EVs in terms of their nanometer size, cellular targeting, and low immunogenicity make them excellent candidates for next-generation drug-delivery systems.

## Conclusion and Perspectives

There are some unresolved issues regarding Mφ-EVs, such as the efficiency of their isolation and purification (195). To address this, Jang et al. (196) created bioinspired exosome-mimetic nanovesicles by breaking down monocytes or Mφ and found that they had similar functional properties as EVs, with 100-fold better isolation and purification efficiency; they also induced TNF-α-stimulated endothelial cell death and showed antitumor activity *in vivo*. Choo et al. (175) prepared exosome-mimetic nanovesicles derived from M1Mφ, which effectively repolarized M2Mφ to M1Mφ and promoted the antitumor efficacy of programmed death ligand 1. EVs have been characterized based on protein content (23, 197). However, the molecular hallmarks specifically distinguishing each EV subtype remain unclear (23). This issue should be further explored and addressed.

Overall, studies have shown that Mφ-EV-based immuno-modulation strategies are effective treatments for various pathological conditions. In the future, more studies are needed to further investigate Mφ-EV-related mechanisms and develop Mφ-EVs based on therapeutic strategies.

## Author Contributions

QY and YHZ initiated the project, made suggestions and revised the article. YX searched the database and wrote the first draft of the manuscript. XS, YD, MW, and YMZ revised and finalized the manuscript. All authors contributed to the article and approved the submitted version.

## Funding

This work was supported by National Natural Science Foundation of China (82072435, 81871782), Tianjin Science Fund for Distinguished Young Scholars (18JCJQJC47900), and Tianjin Science and Technology Program (20JCYBJC01440).

## Conflict of Interest

The authors declare that the research was conducted in the absence of any commercial or financial relationships that could be construed as a potential conflict of interest.

## Publisher’s Note

All claims expressed in this article are solely those of the authors and do not necessarily represent those of their affiliated organizations, or those of the publisher, the editors and the reviewers. Any product that may be evaluated in this article, or claim that may be made by its manufacturer, is not guaranteed or endorsed by the publisher.
